# Supplementary material for: TBC1D14 regulates autophagy via the TRAPP complex and ATG9 traffic
Source: EMBO J. 2015 Dec 28;35(3):281–301. doi: 10.15252/embj.201592695 (PMC4741301; doi:10.15252/embj.201592695)
Supplement: Supplementary file 1 — Appendix [file EMBJ-35-281-s001.pdf]

Appendix

Contents

Page 2 – Supplementary Materials and Methods
Page 7 – Appendix Table S1
Page 8 – Figure Legend for Appendix Figure S1
Page 9 – Appendix Figure S1

Supplementary materials and methods

Cell culture

HEK293A, GFP-LC3-HEK293A and 9B9 culture conditions have been described elsewhere (Chan et al., 2007, Chan et al., 2009, Orsi et al., 2012). GFP-DFCP1-HEK cells were maintained in DMEM + 10% FBS + 4 mM glutamine (DMEM) + 400 ug/ml G418 and were a gift from N. Ktistakis (Axe et al., 2008). HeLa-C1 cells were maintained in DMEM + 1 µg/ml puromycin (Sigma-Aldrich) and were a gift from A. Peden (Gordon et al., 2010). HEK293T cells were lentivirally transduced with viruses encoding a control shRNA (shCtrl, RHS4346, GE Healthcare) or shRNA targeting TRAPPC8 (398843, GE Healthcare) and maintained in DMEM + 1 µg/ml puromycin.

Antibodies

Anti-Beta tubulin (rabbit polyclonal) and LC3-B (rabbit polyclonal) antibodies were from Abcam. Anti-actin AC40 (mouse monoclonal), ERGIC53 (rabbit polyclonal) and TRAPPC8 (rabbit polyclonal) were from Sigma-Aldrich. Anti-TRAPPC4 and TRAPPC12 (mouse polyclonal) were from Abnova. Anti-ULK1, GFP and RAB1B (rabbit polyclonal) were from Santa Cruz Biotech. Anti-RAB11A (rabbit monoclonal) was from Life Technologies. Anti-GM130 (mouse monoclonal) was from Beckton-Dickinson. Recombiant human anti RAB1B-GTP (ROF7) was from Adipogen. Anti S6 ribosomal protein (rabbit monoclonal) and anti Phospho-S6 (Ser240/244) were from Cell Signalling Technology. Anti-TBC1D14 (rabbit polyclonal) (Longatti et al., 2012), anti mATG9 rabbit polyclonal (Young et al., 2006), anti-WIPI2 mouse monoclonal (Polson et al., 2010) and anti mATG9 Armenian hamster monoclonal (Webber & Tooze, 2010) have been previously described. Mouse monoclonal anti GFP (3E1) and myc (9E10) were from the Francis Crick Institute.

siRNA

All siRNA duplexes were from Dharamacon/GE Healthcare. RAB1A and B smartpools (L-008283-00, L008958-01), RISC free (D-001220-01) TBC1D14 (J-014032-12), TRAPPC4 (D-020929-01), TRAPPC8 (J-010645-19), ULK1 (D-005049-03)

Immunoprecipitation

10 cm tissue culture plates of cells (approximately 8 x 10^6 cells) were washed twice with PBS, harvested and lysed in 400 µl TNTE (50 mM Tris HCL pH 7.4, 150 mM

NaCl, 5 mM EDTA, 1% Triton X-100) supplemented with EDTA-free protease inhibitor cocktail and Phos-Stop (PI) (Roche). For GFP-Trap IP, the clarified lysates were incubated with blocked agarose beads (Chromotek) at 4 °C for 30 minutes, then with GFP-Trap beads for 60 minutes at 4 °C. For endogenous IP, 30 µl protein-G sepharose (Sigma-Aldrich) was incubated with 3 µg mouse anti TTC15/TRAPPC12 (Abnova) or 3 µg mouse anti-GFP (3E1; Francis Crick Institute) in PBS + 0.01% Triton X-100 for one hour, then washed in TNTE+PI to remove excess antibodies. The clarified lysate was incubated with 30 ul protein G-sepharose to pre-clear the lysate for half an hour, then with the antibody-bead complexes for two hours at 4 °C. In both cases, the beads were washed three times with lysis buffer, then resuspended in 30 µl 2 x Laemmli Sample Buffer (LSB) and subjected to SDS-PAGE.

For figure EV2E, HEK293A cells overexpressing myc-TBC1D14 or transfected with empty vector (pcDNA3.1) and co-transfected with plasmids encoding either GFP or GFP-Golgin 84 were lysed in RAB lysis buffer (20 mM Na HEPES ph 7.4, 150 mM NaCl, 5 mM MgCl2, 1 mM DTT, 0.3% Triton X-100 plus PI), subject to GFP-Trap IP and immunoblotted for GFP, RAB1B and myc. The same conditions were used in Figure EV2F, with HEK293A cells transfected with the indicated siRNA duplexes and co-transfected with GFP-Golgin 84 and blotting for GFP, RAB1B, TBC1D14 and TRAPP subunits.

BioID analysis

An adapted version of the original Bio ID protocol (Roux et al., 2012) was used to identify TRAPP subunits proximal to TBC1D14. 4 x 15 cm dishes (approximately 20 million cells/plate) of HEK293A cells were transfected with myc-BioID or myc-BioID–TBC1D14. After transfection, the cells were recovered in DMEM + 50 µM Biotin. The following day, the cells were harvested in PBS and lysed in 1.5 ml TNTE + PI and centrifuged to pellet insoluble components. The lysates were split and denatured at room temperature with 1% SDS (D) or not, by adding an equivalent volume of TNTE + PI (ND). The lysates were incubated with streptavidin-agarose (Pierce) for 1 hour at room temperature to precipitate biotinylated proteins. Bound proteins were eluted in 2x LSB + 3 mM Biotin. For mass spectrometry analysis, the samples were subjected to SDS-PAGE, stained with colloidal Coomassie (Invitrogen), lanes excised and analysed by mass spectrometry. For Western blot

analysis, the eluted proteins were blotted with anti-myc, streptavidin-HRP and TRAPP antibodies.

Mass spectrometry analysis

For mass spectrometry analysis, the samples were subjected to SDS-PAGE, stained with colloidal Coomassie (Invitrogen) and eight bands covering the entire molecular weight range were excised. In-gel trypsin digestion was performed overnight at 37 °C using a Perkin Elmer Janus liquid handling system. Peptide extracts were acidified to 0.1% TFA and subjected to LC-MS analysis using an Ultimate3000 nano HPLC system connected to a Q-Exactive mass spectrometer (Thermo Scientific). The instrument was operated in a top 10 data dependent acquisition mode. Raw mass spectrometry data was processed using MaxQuant version 1.3 (Cox & Mann, 2008). Data was searched against a Uniprot FASTA database containing human sequences and intensity based absolute quantification (iBAQ) (Schwanhausser et al., 2011) was used for label free quantification. iBAQ values were $\log_{10}$ transformed and missing values were imputed by drawing from a random distribution using default settings (width 0.3, down shift 1.8) in Perseus software version 1.4.0.2. iBAQ scatter plots were visualised using R software.

Size exclusion chromatography

For figure EV2D, approximately 2 × 107 HEK293T cells were homogenised using a syringe and needle in 500 µl homogenisation buffer, the nuclei removed by 2 × 3 minute centrifugations at 2500 rcf and 4 °C, and membranes removed by ultracentrifugation for 1 hour at 100,000 rcf. The resulting supernatant was used in size exclusion chromatography with a Superose 6 column (GE Healthcare), with an elution volume of 1.2 column volumes and collecting 0.5 ml fractions. Proteins from fractions were concentrated using 10 µl Strataclear beads (Stratagene) and bound proteins eluted in 30 µl 2 x LSB. Fractions 19-40 were subjected to immunoblot analysis with the indicated antibodies, alongside 1% of the input.

Protein purification

Plasmids encoding GST and GST-TBR (pGEX 4T1 (GE Healthcare) and pGEX 4T1-TBR) were transformed into BL-21-DE3 *E. coli* (Agilent). The bacteria were grown in 2 l 2YT (1.6 % tryptone, 1 % yeast extract, 0.5 % NaCl) to logarithmic phase, and protein expression induced with 1 mM IPTG for 4 hours at 37 °C. The bacteria were

harvested by centrifugation, the pellets resuspended in PBS + 1% Triton X-100 and treated with 1 mg/ml lysozyme for 30 minutes on ice. The resulting suspension was sonicated to break the cells and insoluble components pelleted by centrifugation at 20 000 rcf for 30 minutes. The resulting supernatant was incubated with glutathione Sepharose 4B resin (GE Healthcare) for one hour at 4 °C, and the resin washed with PBS + 1% Triton X100, PBS + 0.5M NaCL and PBS. The GST tagged proteins were eluted from the beads using 50 mM Tris-HCl pH 8 + 10 mM reduced glutathione.

For insect cell expression, 100 ml of SF9 cells were infected with baculovirus encoding GST or GST-TBC1D14 for 72 hours at an MOI of 2. The cells were resuspended in 10 ml ice cold Buffer A (50 mM Tris, pH 7.5 150 mM NaCl, 1 mM EDTA, 0.1 % (v/v) Triton X-100, 5% (v/v) glycerol, 1 mM DTT, 10 mM Benzamidine, 1 mM NaF, 10 mM β-glycerophosphate + protease inhibitors) and sonicated to break open cells. The lysate was clarified by centrifugation at 20 000 rcf for 15 minutes, and then incubated with glutathione-Sepharose 4B resin for 2 hours at 4 °C. The resin was washed four times in buffer A and used in pull-down assays.

References

Axe EL, Walker SA, Manifava M, Chandra P, Roderick HL, Habermann A, Griffiths G, Ktistakis NT (2008) Autophagosome formation from membrane compartments enriched in phosphatidylinositol 3-phosphate and dynamically connected to the endoplasmic reticulum. *J Cell Biol* 182: 685-701

Chan EY, Kir S, Tooze SA (2007) siRNA screening of the kinome identifies ULK1 as a multidomain modulator of autophagy. *J Biol Chem* 282: 25464-74

Chan EY, Longatti A, McKnight NC, Tooze SA (2009) Kinase-inactivated ULK proteins inhibit autophagy via their conserved C-terminal domain using an Atg13-independent mechanism. *Mol Cell Biol* 29: 157-171

Cox J, Mann M (2008) MaxQuant enables high peptide identification rates, individualized p.p.b.-range mass accuracies and proteome-wide protein quantification. *Nat Biotechnol* 26: 1367-72

Gordon DE, Bond LM, Sahlender DA, Peden AA (2010) A targeted siRNA screen to identify SNAREs required for constitutive secretion in mammalian cells. *Traffic* 11: 1191-204

Longatti A, Lamb CA, Razi M, Yoshimura S-I, Barr FA, Tooze SA (2012) TBC1D14 regulates autophagosome formation via Rab11 and recycling endosomes. *J Cell Biol* 197: 659-675

Orsi A, Razi M, Dooley H, Robinson D, Weston A, Collinson L, Tooze S (2012) Dynamic and transient interactions of Atg9 with autophagosomes, but not membrane integration, is required for autophagy. *Mol Biol Cell* 23: 1860-1873

Polson HEJ, de Lartigue J, Rigden DJ, Reedijk M, Urbe S, Clague MJ, Tooze SA (2010) Mammalian Atg18 (WIPI2) localizes to omegasome-anchored phagophores and positively regulates LC3 lipidation. *Autophagy* 6: 506-522

Roux KJ, Kim DI, Raida M, Burke B (2012) A promiscuous biotin ligase fusion protein identifies proximal and interacting proteins in mammalian cells. *J Cell Biol* 196: 801-10

Schwanhausser B, Busse D, Li N, Dittmar G, Schuchhardt J, Wolf J, Chen W, Selbach M (2011) Global quantification of mammalian gene expression control. *Nature* 473: 337-42

Webber JL, Tooze SA (2010) Coordinated regulation of autophagy by p38alpha MAPK through mAtg9 and p38IP. *EMBO J* 29: 27-40

Young ARJ, Chan EYW, Hu XW, Köchl R, Crawshaw SG, High S, Hailey DW, Lippincott-Schwartz J, Tooze SA (2006) Starvation and ULK1-dependent cycling of Mammalian Atg9 between the TGN and endosomes. *J Cell Sci* 119: 3888-3900

**TRAPP complex subunit**	**Alternative names**	**Unique peptide number**	**Mascot Score**	***S. cerevisiae* orthologue**
TRAPPC8	KIAA1012	10	462	Trs85
TRAPPC12	TTC15	6	287	Metazoan-specific
TRAPPC3	mBET3	4	181	Bet3
TRAPPC1	BET5, MUM2	1	49	Bet5
TRAPPC6B	-	1	40	Trs33
TRAPPC2L	Sedlin-like, HSPC126	1	21	Trs20

Appendix Table S1. TRAPP subunits identified by mass spectrometry (relating to Figure 1A)

Appendix Figure S1. D/D solubiliser treatment does not inactivate mTOR or induce autophagy. A) HeLa C1 cells were treated with D/D solubiliser (D/D) Rapamycin (Rapa), Torin or maintained in full medium for 80 minutes. The cells were harvested and lysates immunoblotted for GFP, Beta-tubulin, S6 ribosomal protein and phospho-S6 (Ser240/244). Bar charts show percentage of GFP-FM4-hGH remaining in the cells normalised to tubulin and levels of phospho-S6 normalised to total S6 and are the quantification of three independent experiments ± s.e.m. **** $p<0.001$, one-way ANOVA followed by Dunnett's multiple comparison test, n.s. – not statistically significant. **B)** HEK293A cells were treated as in (A), with the addition of bafilomycin A1 (BafA1). Lysates were immunoblotted for LC3B and tubulin as a loading control. Bar chart is the quantification of three independent experiments ± s.e.m. ** $p<0.01$, ***$p<0.001$, one way ANOVA followed by Sidak's multiple comparison test, n.s. – not statistically significant.

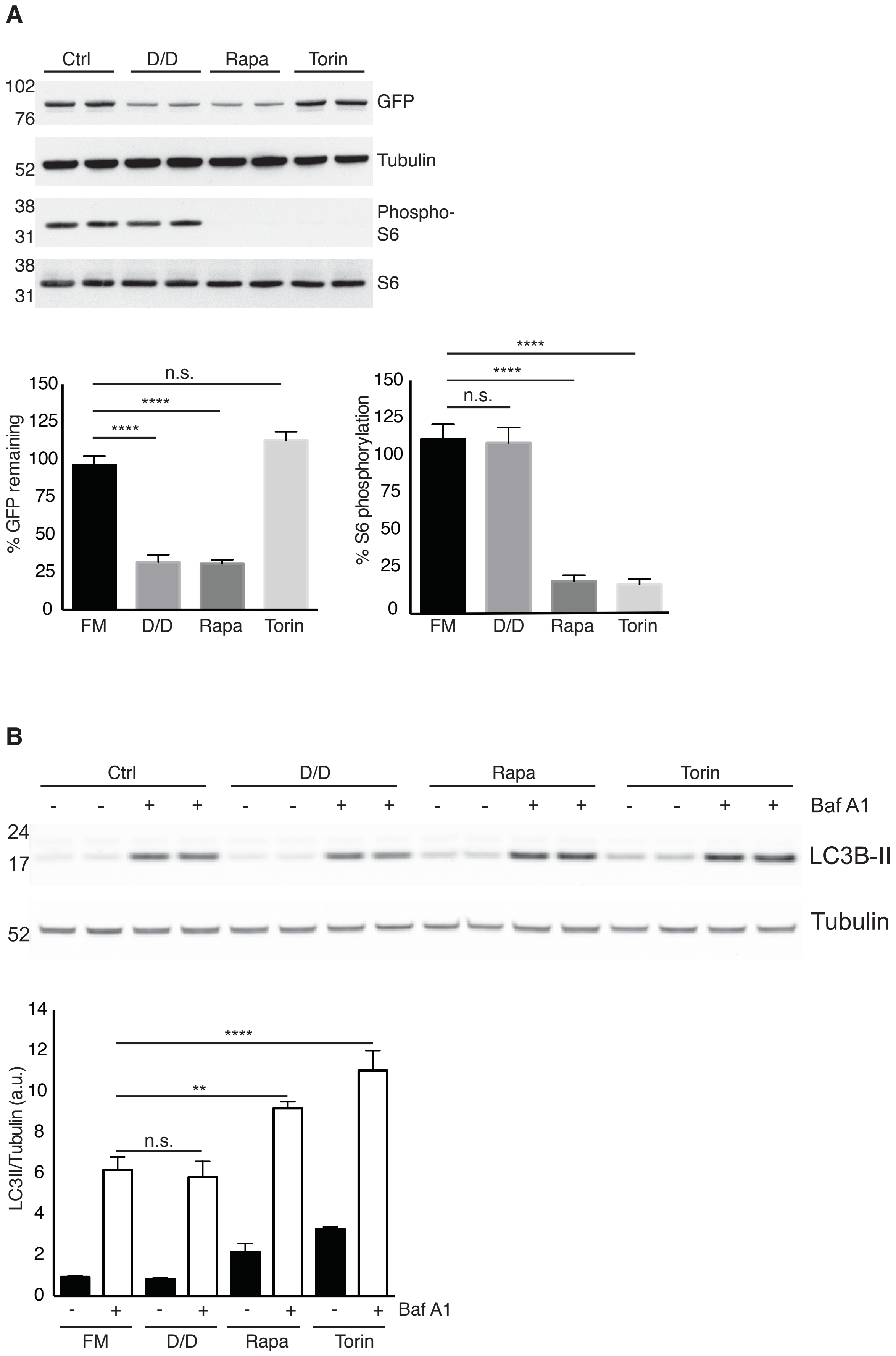

Appendix Figure S1
