# Supplementary material for: TBC1D14 regulates autophagy via the TRAPP complex and ATG9 traffic
Source: EMBO J. 2015 Dec 28;35(3):281–301. doi: 10.15252/embj.201592695 (PMC4741301; doi:10.15252/embj.201592695)
Supplement: Supplementary file 2 — Expanded View Figures PDF [file EMBJ-35-281-s002.pdf]

Expanded View Figures

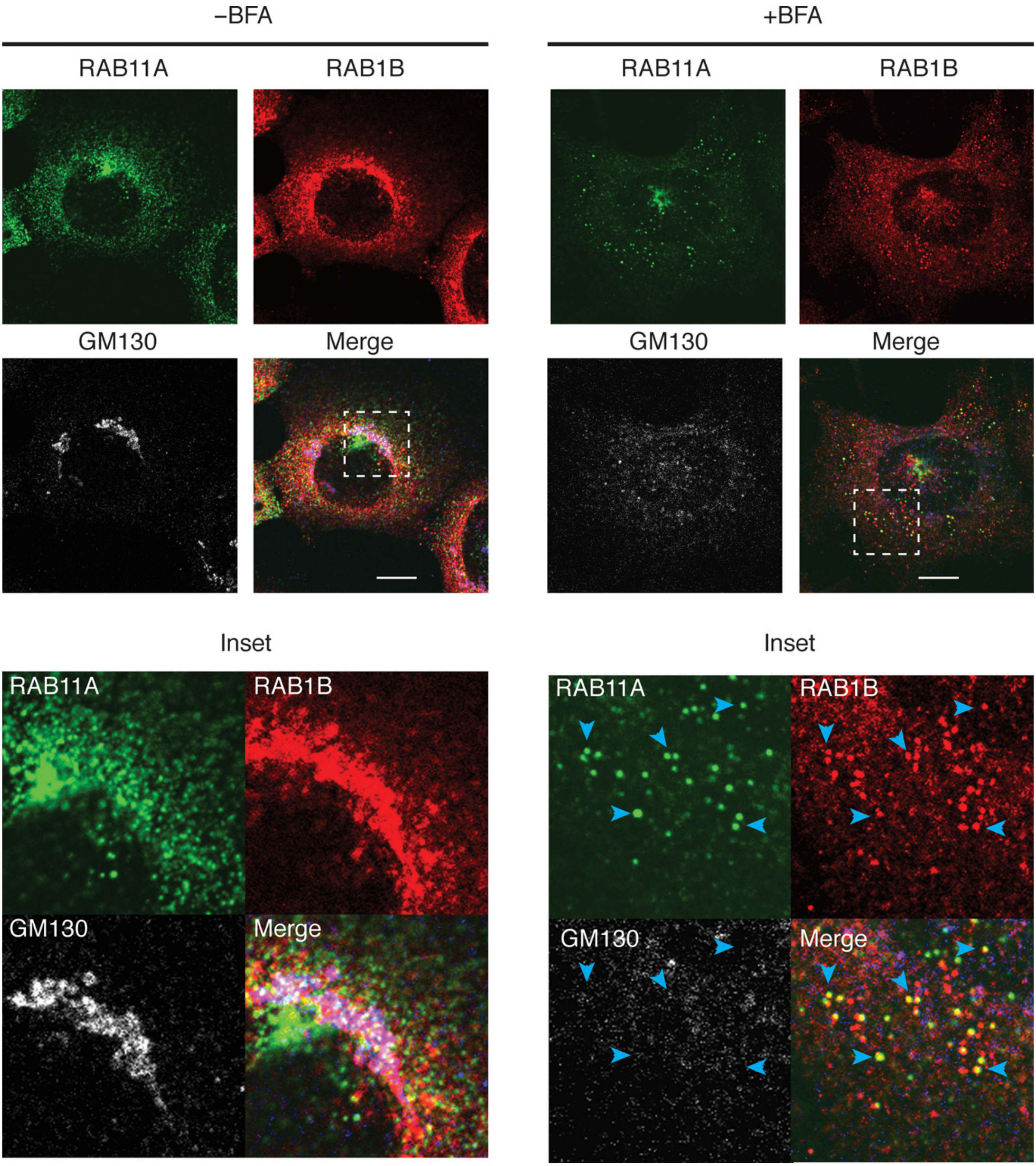

Figure EV1. Co-localisation of endogenous RAB1B and RAB11A.

HEK293A cells were treated with 10 μg/ml BFA for 30 min (+ BFA) or not (−BFA), fixed and stained for RAB11A (green), RAB1B (red) and GM130 (white, blue in merge). Scale bars, 10 μm. Blue arrowheads indicate RAB1B- and RAB11A-positive structures—note the absence of the early Golgi marker GM130 on these puncta.

 © 2015 The Authors

Figure EV2. Identification of an endogenous TRAPPIII-like complex in mammalian cells.

A Scatterplot of $\log_{10}$-transformed iBAQ values for myc-BioID versus myc-BioID-TBC1D14 non-denaturing pulldowns analysed by mass spectrometry.

B Scatterplot of $\log_{10}$-transformed iBAQ values for myc-BioID-TBC1D14 denaturing versus myc-BioID-TBC1D14 non-denaturing pulldowns analysed by mass spectrometry.

C HEK293A cells (1 × 10 cm plate per condition) were lysed in 400 μl TNTE and subjected to immunoprecipitation with mouse anti-TRAPPC12/TTC15 or mouse anti-GFP bound to protein G-Sepharose. The bound proteins were immunoblotted for TRAPPC8 and TRAPPC12.

D TRAPPC8, TRAPPC12 and the core subunit TRAPPC4 co-elute in a complex of approximately 600 kDa. Blots are representative of three independent experiments. Approximate elution profiles of molecular weight standards are indicated above the fractions.

E TBC1D14 overexpression does not promote GTP binding to RAB1B. Bar chart is quantification of the percentage of RAB1B precipitated (normalised to GFP-Golgin-84) upon myc-TBC1D14 overexpression compared to empty vector expression from three independent experiments. n.s. = not significant, unpaired *t*-test.

F Lysates of HEK293A cells expressing GFP-Golgin-84 and transfected with RISC-free control siRNA or siRNA targeting TBC1D14, TRAPPC8 or RAB1B were subjected to GFP-Trap IP as in (E) and immunoblotted for RAB1B, TBC1D14, TRAPPC8, TRAPPC4 and GFP. Bar chart is quantification of the percentage of RAB1B precipitated (normalised to GFP-Golgin-84) upon myc-TBC1D14 overexpression compared to RISC-free siRNA from three independent experiments. $^{**}P < 0.01$, $^{***}P < 0.001$, one-way ANOVA followed by Sidak's multiple comparison test.

© 2015 The Authors

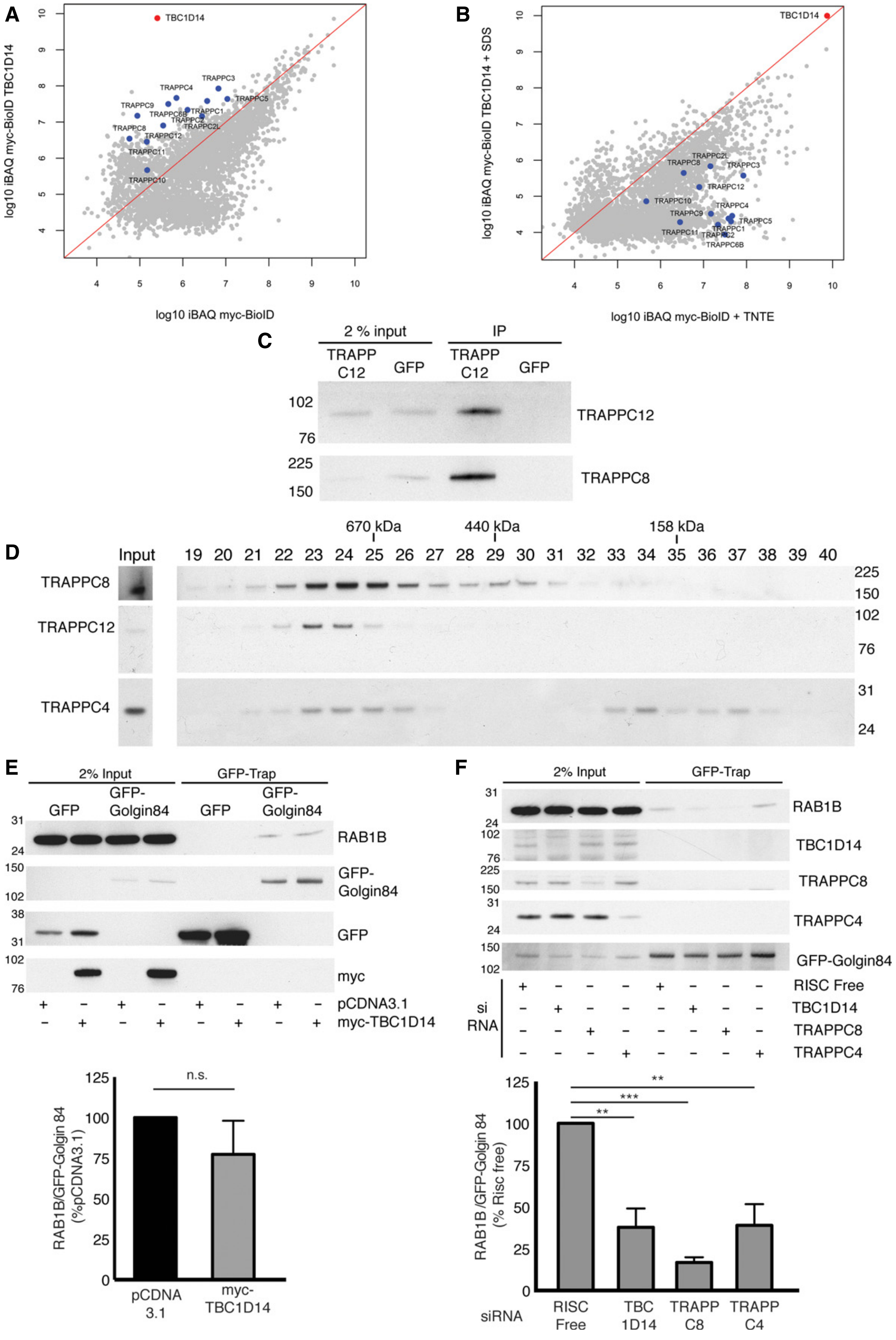

Figure EV2.

 © 2015 The Authors

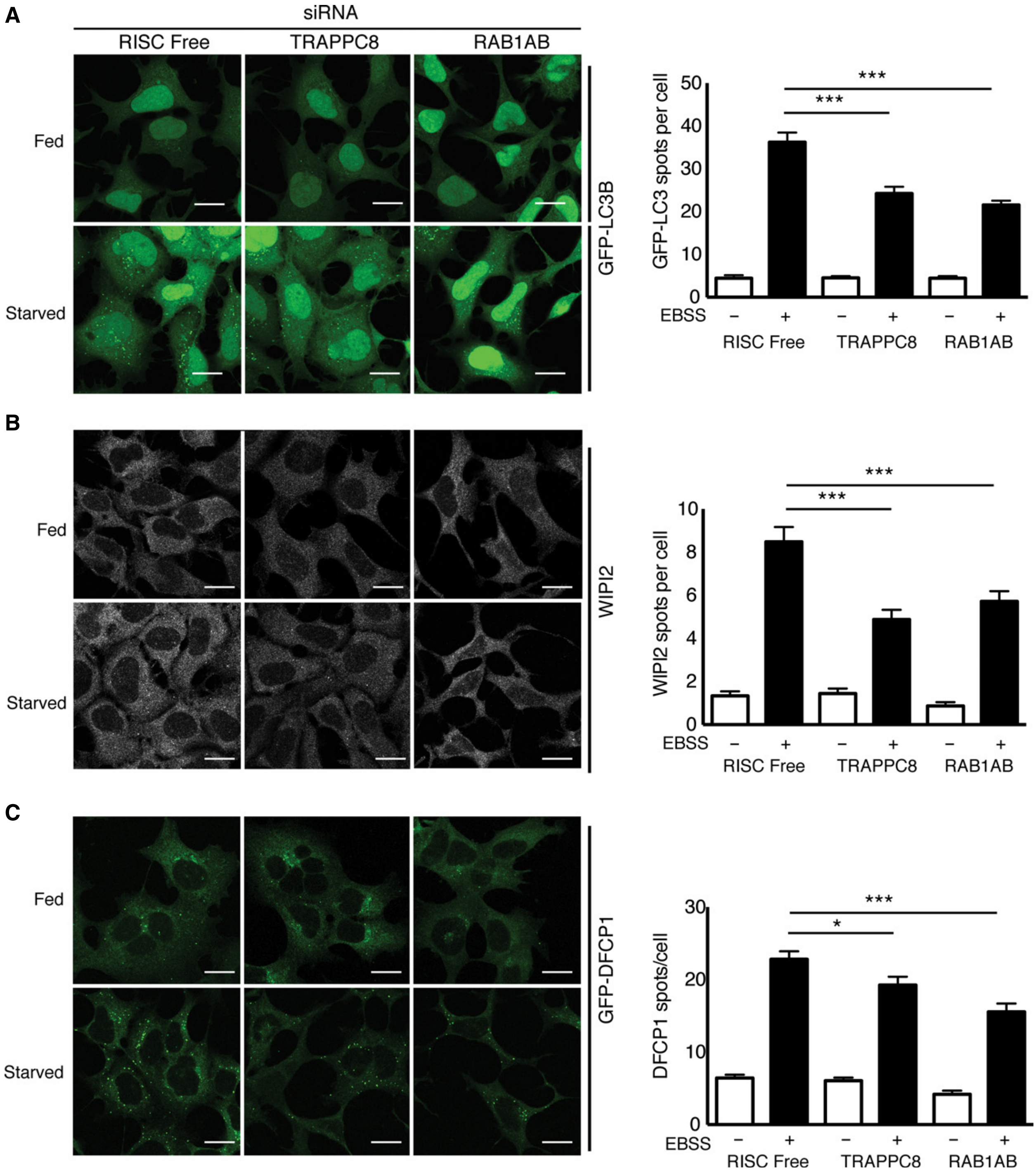

Figure EV3. Depletion of TRAPPC8 or RAB1 affects autophagosomal punctum formation at an early step.

A HEK293A cells stably expressing GFP-LC3B (green) were transiently transfected with RISC-free control siRNA or siRNA duplexes targeting TRAPPC8 or RAB1A and B then treated with EBSS (starved) or not (fed), fixed and analysed by confocal microscopy.

B HEK293A cells were transiently transfected with RISC-free control siRNA or siRNA duplexes targeting TRAPPC8 or RAB1A and B then treated with EBSS (starved) or not (fed), fixed, stained for WIPI2 (white) and analysed by confocal microscopy.

C HEK293A cells stably expressing GFP-DFCP1 (green) were transiently transfected with RISC-free control siRNA or siRNA duplexes targeting TRAPPC8 or RAB1A and B then treated with EBSS (starved) or not (fed), fixed and analysed by confocal microscopy.

Data information: 10 confocal images were taken of each condition and the number of autophagic puncta per cell enumerated using Imaris software (Bitplane). Scale bars, 20 μm. Bar charts represent the mean of three experiments, ± s.e.m *$P < 0.05$, ***$P < 0.001$.

© 2015 The Authors

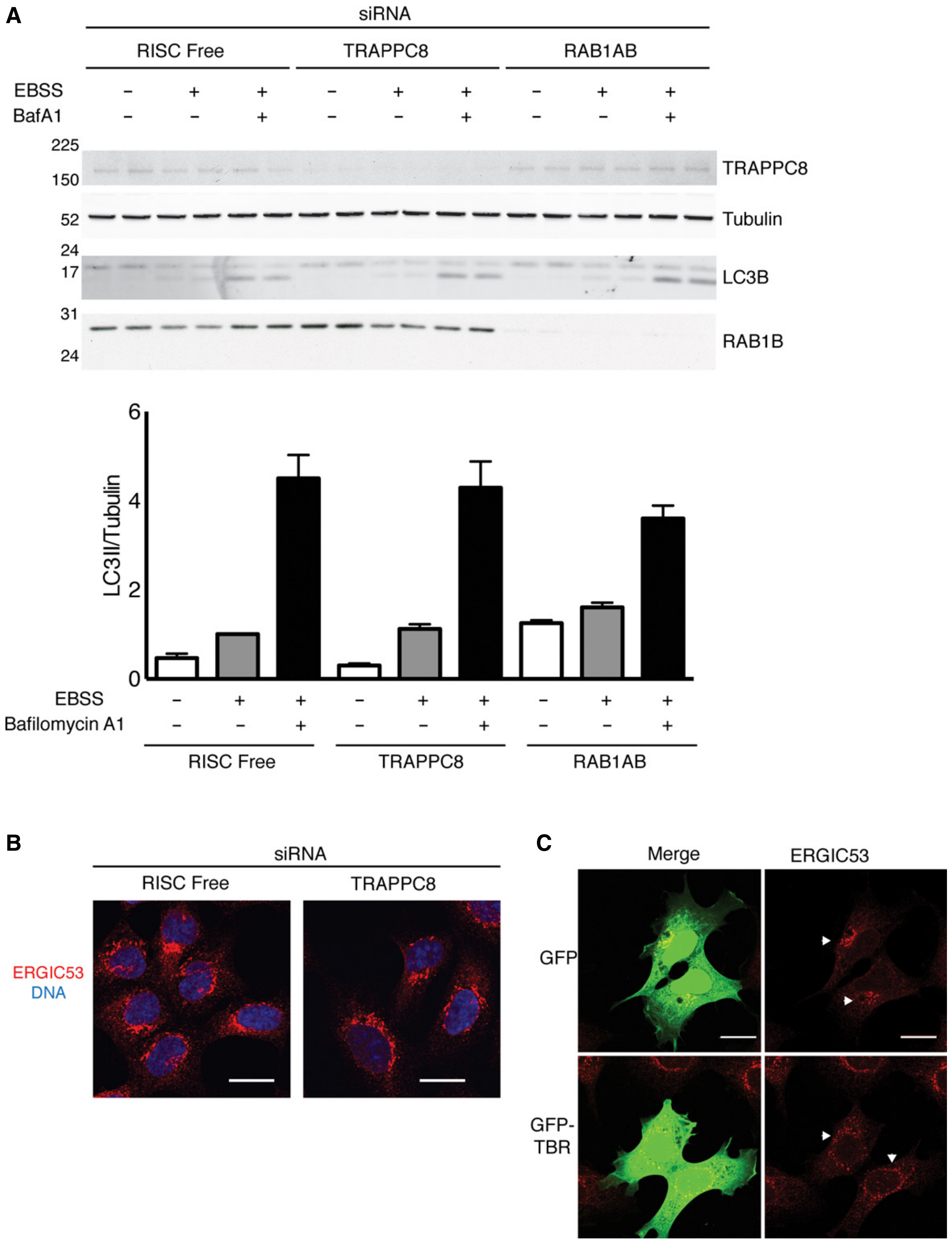

Figure EV4. Depletion of TRAPPC8 does not affect LC3 lipidation, and RAB1 depletion has a mild effect.

A HEK293A cells transiently transfected with control RISC-free control siRNA or siRNA duplexes directed against TRAPPC8 or RAB1AB were treated with EBSS, EBSS + 100 nM bafilomycin A1 or not for 2 h, lysed and subjected to immunoblotting for LC3B, tubulin, TRAPPC8 and RAB1B. The amount of LC3B/tubulin for each condition from three independent experiments is shown in the bar graph, ± s.e.m.

B Cells depleted for TRAPPC8 or not (RISC free) were immunostained for ERGIC53 (red) and Hoechst (DNA; blue). Scale bars, 20 μm.

C Cells expressing GFP or GFP-TBR (green) were immunostained for ERGIC53 (red; arrowheads indicate transfected cells) and analysed by confocal microscopy. Scale bars, 20 μm.

 © 2015 The Authors

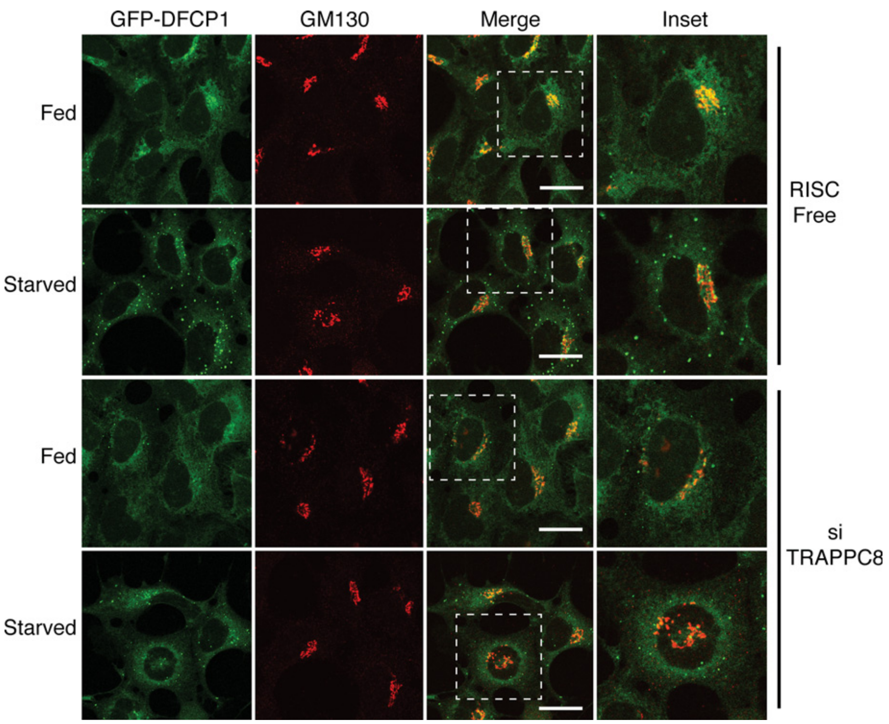

Figure EV5. Depletion of TRAPPC8 results in mis-localisation of GFP-DFCP1.
HEK293A cells stably expressing GFP-DFCP1 (green) were transiently transfected with control siRNA or siRNA targeting TRAPPC8, starved in EBSS for 2 h (starved) or not (fed), fixed, stained for GM130 (red) and analysed by confocal microscopy. Scale bars, 20 μm.

© 2015 The Authors
